# The psychological journey of weight gain in psychosis

**DOI:** 10.1111/papt.12386

**Published:** 2022-02-08

**Authors:** Felicity Waite, Amy Langman, Sophie Mulhall, Margaret Glogowska, Jamie Hartmann‐Boyce, Paul Aveyard, Belinda Lennox, Oxford Cognitive Approaches to Psychosis Patient Advisory Group, Thomas Kabir, Daniel Freeman

**Affiliations:** ^1^ Department of Psychiatry University of Oxford Oxford UK; ^2^ Oxford Health NHS Foundation Trust Oxford UK; ^3^ Nuffield Department of Primary Care Health Sciences University of Oxford Oxford UK; ^4^ McPin Foundation London UK

**Keywords:** confidence, delusions, obesity, schizophrenia, treatment, voices, weight loss

## Abstract

**Background:**

Rapid weight gain is common with antipsychotic medication. Lost confidence, low mood and medication non‐adherence often follow. Yet, the dynamic interactions between the physical and psychological consequences of weight gain, and implications for intervention, are unknown.

**Objectives:**

We examined first‐person accounts of weight gain to identify preferences for weight change interventions.

**Design:**

A qualitative design was used to explore patients’ experiences of weight change in the context of psychosis.

**Method:**

Semi‐structured interviews, analysed using grounded theory, were conducted with 10 patients with psychosis. Sample validation was conducted with peer researchers with lived experience of psychosis.

**Results:**

Patients described that initially the extent and speed of weight gain was overshadowed by psychotic experiences and their treatment. This led to a shocking realisation of weight gain. The psychological impact of weight gain, most strikingly on the self‐concept, was profound. Loss of self‐worth and changed appearance amplified a sense of vulnerability. There were further consequences on mood, activity and psychotic experiences, such as voices commenting on appearance, that were additional obstacles in the challenging process of weight loss. Sedative effects of medication also contributed. Unsuccessful weight loss left little hope and few preferences for interventions. Early information about common weight gain trajectories and working with experts‐by‐experience were valued. Rebuilding self‐confidence, efficacy and worth may be a necessary first step.

**Conclusions:**

The journey of weight gain in patients with psychosis is characterised by loss of self‐worth, agency and hope. There are multiple stages in the journey, each with different psychological reactions, that may need different treatment responses.

**Practitioner points**

- Many patients with psychosis experience rapid weight gain, with significant consequences for both physical and mental health.
- Participants in this study described a journey of weight gain characterised by multiple stages each with differing psychological reactions.
- Different treatment responses may be required at different stages of the journey, with a focus on rebuilding self‐confidence as a potentially necessary first step.
- Participants shared preferences for treatment that included early information about common weight gain trajectories and the potential role of working with experts‐by‐experience as well as health professionals.

## BACKGROUND

Rapid weight gain in the first phase of antipsychotic medication use is common (Álvarez‐Jiménez et al., 2008; Pillinger et al., 2020; Zipursky et al., 2005). The excess weight persists. More than 50% of patients with psychosis have a BMI in the obese range (Annamalai et al., 2017). This is double the rate for adults in the general population (ONS, 2017). The physical consequences are clear: excess weight contributes to a reduction in life expectancy for patients with psychosis (Hayes et al., 2017). Excess weight in patients with psychosis is associated with medication non‐adherence, social isolation, poor quality of life and low self‐esteem (Mccloughen & Foster, 2011). Arguably, these effects may also make psychotic experiences more likely to occur.

To date, weight loss interventions, including specific programmes designed for patients with psychosis, have shown limited clinical benefits for patients with psychosis (Álvarez‐Jiménez et al., 2008; Brown et al., 2018; Naslund et al., 2016; Speyer et al., 2019). Greater effectiveness is apparent for interventions that make adaptions for the patient group, for example, providing practical tools such as pedometers or interim support between sessions (Lee et al., 2021). Treatment improvement will likely benefit from understanding the potential interaction between the physical and psychological consequences of excess weight in patients with psychosis.

Grounded theory is a qualitative method used to develop a model, generate further hypotheses and inform intervention development (Charmaz, 2014). In this study, our aim was to gain an understanding of the experience of weight gain and preferences for weight management interventions in patients with psychosis.

### Design

This was an exploratory qualitative grounded theory study utilising semi‐structured interviews to explore weight changes and preferences for weight management interventions in patients with psychosis. Criteria to ensure quality in the conduct of qualitative research (Barbour, 2001; Tong et al., 2007; Yardley, 2008) were used to inform the design and ensure rigour in the conduct of the study. The study had full ethical approval from the NHS Health Research Authority (HRA) (ref. 19/SC/0255).

## METHODS

### Research team

The research was primarily conducted within a clinical research group interested in understanding and developing psychological treatments for distressing psychotic experiences, working alongside an advisory group of people with lived experience of psychosis. The advisory group were involved throughout the study, including in the design, analysis and write‐up. The research team have expertise in the development and delivery of psychological models and therapy, treatment of early psychosis, behavioural medicine, weight management programmes and in qualitative methodology.

### Participants

Participants were recruited from local clinical services in Oxford Health NHS Foundation Trust. This included community mental health and early intervention in psychosis services. The inclusion criteria were as follows: aged 16 years and above and a primary diagnosis of non‐affective psychosis (e.g., schizophrenia, schizoaffective disorder, schizophreniform disorder, delusional disorder, brief psychotic disorder and psychotic disorder not otherwise specified). Participants without capacity to consent, insufficient English language comprehension (i.e., unable to comprehend the consent form or participate in the interview without an interpreter) or primary diagnoses of alcohol or drug misuse; personality disorder; learning disability or with significant forensic history or significant current risk to self or others were excluded. Potential participants were identified by members of their clinical team. If consent was provided, screening was conducted by the study team (AL or SM). Of the 16 people approached, three people declined, three people agreed to be contacted at a later date and 10 people agreed to participate. Written informed consent, including consent to the use of pseudonymised quotes, was obtained.

#### Purposive sampling

Purposive sampling was used to ensure participants had a diverse range of experiences of weight changes, including people who had, or had not, experienced significant weight gain, and people who had attempted weight loss and been successful or unsuccessful in these attempts. In response to the emerging findings, participants were theoretically sampled to investigate initial categories. Variation across gender, age, relationship status, social support, physical health condition, expressed motivation in managing weight and recovery stage was also sought.

### Procedure

#### Interview guide – iterative development and use

The semi‐structured interview guide was developed in consultation with people with lived experience of psychosis, with reference to existing literature and in line with the study aims. The guide included four topics: experience of weight change, impact of weight, satisfaction and effort to change weight and preferences for weight change interventions (see Appendix S1). AL conducted one pilot interview to increase familiarity with the guide and generate additional open‐ended probe questions to encourage participants to expand their commentary. The guide was used flexibly with the focus on eliciting the participants’ own account. Whenever possible, the participants own language was used. In line with the iterative data collection and analysis process, the topic guide was adapted in light of emerging categories. For example, it became clear that participants were unsure what the possibilities for intervention or prevention might include. Therefore, cue cards (incorporating prompts regarding treatment options and potential motivators) were introduced to facilitate discussion and idea generation.

#### Interview process

Interviews were conducted between 29 July 2019 and 5 February 2020 by AL (*n* = 8) or SM (*n* = 2) at participants’ homes (*n* = 8) or their local mental health clinic (*n* = 2), depending on participant preference. There were no prior relationships between the researcher conducting the interviews and the participants. To calculate BMI, height and weight measurements were taken at the interview. The interviews, which lasted 34–82 min (mean = 63.5, *SD* = 13.9), were audio‐recorded and transcribed verbatim by a professional transcriber. During, and immediately after, the interviewer noted initial theoretical categories based upon the participants’ responses.

### Analysis

The driving mechanism behind the constructivist paradigm of grounded theory (Charmaz, 2014) is that the researcher is constantly interacting with the data. In order to account for and limit researcher bias, the core analysis team (AL, SM, FW) maintained a reflexive journal, field notes and memos to track and inform the developing coding framework and record assumptions made throughout the research process. Prior to data collection, a bracketing interview to identify preconceived biases and expectations for the interviews was completed by the two interviewers (AL and SM). To increase reliability, there was double coding for eight interviews, the coding framework was regularly reviewed by the core analysis team (AL, SM, FW) and by the wider team (MG, DF), and adjusted accordingly. Further credibility checking and sample validation was conducted with people with lived experience of psychosis during the latter stages of the analysis process.

The analytic procedure for grounded theory, as outlined by Charmaz (2014), was as follows. This included: (1) initial line‐by‐line coding of the transcribed interviews, (2) focused coding to form conceptual categories, (3) memo writing to elaborate on processes, assumptions and actions to inform the development of categories and (4) specifying and testing categories through theoretical sampling. This process of constant comparative analysis is iterative and recursive, rather than linear.

Details regarding each code were recorded to form a codebook. To facilitate iterative revisions of the framework with later analysis, details of subsumed codes were retained in the codebook. Later in the analytic process, earlier transcripts were reviewed again to ensure pertinent data were captured. In the final stages of analysis, coding checks of each transcript were conducted. The coding framework was revised at each stage of analysis. For example, after coding the first three transcripts, the coding framework highlighted the theme of vulnerability. At first this was identified in relation to psychotic experiences (voices and paranoid fears) and stigma. This was revised to include appearance concerns and later excess weight (physically unable to escape danger and increased judgement from others). The impact on self‐worth was a central category and theoretical insight. It was identified in the first few accounts and then explored in all further accounts. Later analysis revealed the impact on self‐care and the need to regain self‐worth before efforts to lose weight. Earlier transcripts were reviewed again to ensure pertinent data with regards to the importance of regaining self‐worth were captured. Throughout the analytic process, the proposed theoretical framework took a number of variations and revisions. These included: (i) a spiral reflecting the interconnection between the impact of psychosis and weight gain, (ii) separating the effects of psychosis and weight gain to highlight the distinct components of each and (iii) the positioning of self‐concept at the centre with negative impacts from both experiences of early psychosis and weight gain. Sample validation, to contextualise the results of the primary data, was conducted with peer researchers who have lived experience of psychosis. Including a peer perspective can enhance the validity of the analysis as peer researchers bring different insights to the analytic process (Gillard et al., 2010; Sweeney et al., 2013). The peer researchers highlighted the importance of inactivity in addition to changes in food intake, the cycle of hopelessness and despair from regaining weight that had been effortfully lost and the financial burden of weight gain, in particular the need to purchase new clothes.

NVivo version 12 was used to facilitate the coding, organisation and analysis of data.

### Contextualising the data

Ten people participated: three women and seven men. The participants age ranged from 23 to 72 with a mean of 36.6 years (*SD* = 15.2). Two participants were currently in full‐time employment. Eight participants were single. Participants had a range of clinical diagnoses including schizophrenia (*n* = 1), schizoaffective disorder (*n* = 1), acute polymorphic psychotic disorder (*n* = 1) and psychosis not otherwise specified (*n* = 7). Nine participants were currently being prescribed antipsychotic medication. Four participants were smokers. BMI scores ranged from 24 (healthy weight) to 48 (class III severe obesity). All but one of the participants had a BMI in the overweight (*n* = 4) or obese range (*n* = 5). Seven participants were actively trying to lose weight, two (1 M; 1F) were trying to maintain weight loss and one (M) was attempting to gain weight. The main weight management strategies were healthy eating (*n* = 7), exercise (*n* = 5), tracking food intake (*n* = 5) and tracking weight (*n* = 4), with only one participant using a weight loss (diabetes) service.

## RESULTS

**FIGURE 1:**
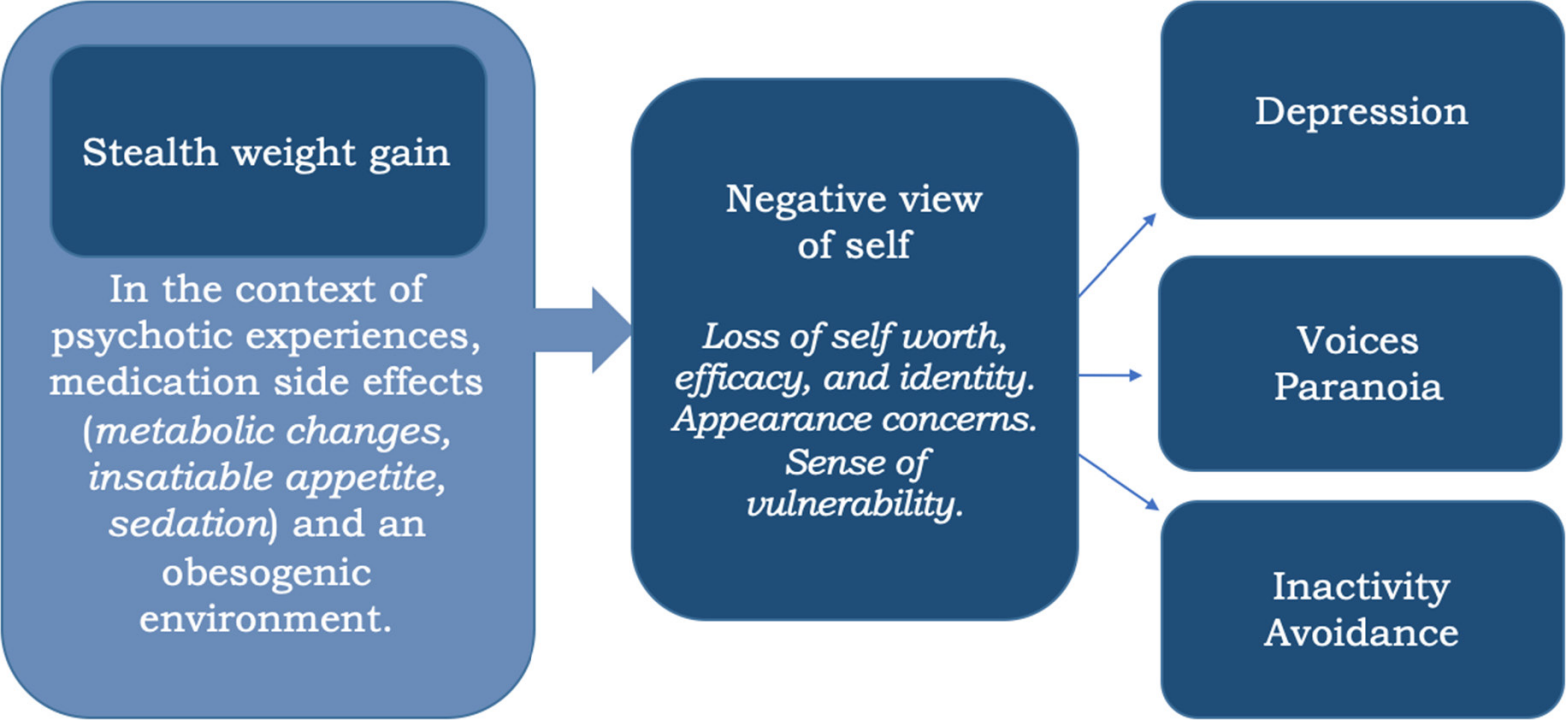
Psychological impact of weight gain in psychosis

Participants described the interplay between psychosis and subsequent weight change as a journey (Table 1). All participants described a destructive impact of psychosis on their lives. This included feeling distressed, preoccupied and vulnerable. Participants described their sense of self as diminished and their lives restricted. Weight gain then compounded many of the same negative consequences and losses, psychologically weighing participants down and leaving them feeling stuck (Figure 1). The route to weight loss began with regaining self‐worth. But momentum was often lost against the hurdles of stigma, medication side effects and ongoing psychotic experiences. Illustrative quotes for each theme are provided in Table S1.

**TABLE 1:** Overview of major themes

Catapulted into obesity Uncontrollable and rapid *– the role of obesogenic medication* Stealth weight gain – *overshadowed by psychosis* Time lag – *shocking realisation* Ground to a halt Losses upon losses Losing self‐worth, losing control and losing myself Amplifies vulnerability Appearance concerns, psychotic experiences and stigma Pulled in two directions – *prioritising mental health* An uphill struggle The first step – regaining self‐worth Heavy load Psychotic experiences Double stigma Depression, withdrawal and rumination – *demotivated and disconnected* Sedative effects of medication Keeping momentum against the gradient – *losing weight is often a losing battle* Support, accountability, agency, hope, personalise and peers Frustration, despair and obesogenic medication.

### Catapulted into obesity

It all felt out of control, all of those feelings felt out of control. Like I didn’t want this, I didn’t decide this. … And it shocked me how it, it just happens. Like someone who is, who would maybe even call themselves fat is not really responsible for it. (P8)

#### Uncontrollable and rapid

Participants described being catapulted in to unwanted obesity. It was an experience of rapid, uncontrollable and unexpected weight gain. Within a few months of starting medication all participants experienced weight gain. The scale of weight gain was striking: more than half the participants gained several stone. One described becoming ‘twice my normal size’ (P2).

Weight gain was predominantly attributed to medication. Either directly, due to metabolic changes or insatiable appetite, or indirectly, by sapping motivation for physical activity and self‐care. There was a striking contrast between those experiencing an insatiable appetite and those who had not changed their food intake, yet were still gaining weight. The similarity in the accounts was the sense of frustration, loss of control and that the explanation was due to medication side effects:

It’s that nothing is enough, just that sensation where you eat but it’s not enough. It’s like you keep eating and that hunger, that sensation of hunger is, is still there. And you eat, you eat but it’s still there and you can tell it’s the medication. (P4)

I mean I wasn’t over eating at all, I was just eating an ordinary, normal amount that I used to before I put on the weight, before I went on the medicine. (P2)

For participant 4, the combination of depression and increased appetite often led to high calorie consumption that fuelled a cycle of weight gain: ‘The reality of the situation makes you more depressed and you stop caring for yourself. But it's also the medication that makes you hungry, that was definitely shocking to me … When I put on extra, I think, the way I was feeling just more, I guess hopeless. Because it's like a chain of events, you know, you are mentally unwell and that will lead for you to make poor choices, stop caring. And that makes me think of I might get diabetes, or I might get heart disease…. Um, so obviously when I got heavier it made me even more depressed. It was like a circle you know’.

Overall, the impact of medication on weight left participants with a feeling of less responsibility for their weight gain, and a sense of being ‘out of control’ both for weight gain and potential weight loss.

It was so irritating for me not to be able to change the fact that I have to take the medicines whether I liked it or not because of my mental health. So, I couldn’t change my mental health or my weight. (P2)

#### Stealth weight gain

The extent and speed of weight gain was initially overshadowed by the onset and treatment of psychotic experiences.

I just wasn’t thinking about anything other than what I was going through with my psychosis…it was what’s going on in my head, in front of me, that was the problem thing, and not what’s happening to my body at the time. (P1)

Most participants were partially aware their weight was increasing. Yet the full extent of weight gain was obscured. Either participants’ attention was consumed by the impact and experience of psychosis (and depression) or they were unconcerned due to absent self‐care or (false) optimism about their ability to lose weight later. For example, for participant 8 the hospital setting allayed his concerns:

It was sort of like [pause] it, it takes time to, to notice the consequences… Every time I got on the scale a number was going up. It just seemed normal…it was just something that was happening…everything is so controlled in hospital…that I didn’t really feel scared for my health in hospital. They were taking my weight every week. That’s why I wasn’t worrying about it in the first place. I was like the body can cope with suddenly gaining a lot of weight. And you know maybe it’s because I’m sitting around in hospital all the time. But [pause] it, I, and then months later I was very aware that I was, er, a very different weight to what I had been. And I would look in the mirror and not like how I looked.

#### Time lag – moment of realisation

For many participants there was a shocking moment of realisation.

I would look at myself in the mirror and sort of not really recognise myself and sort of think, you know, wow, that’s a, that’s a fat person now. (P8)

For all but one participant, the full realisation of weight gain came as they experienced improvement in their psychotic experiences and depression lifted. Discharge from inpatient care was a key trigger for participant 8. For others it was as follows: visible signs such as stretch marks (P7), changed body shape (P8), not fitting into clothes (P1, P2 and P6), decline in physical fitness (P1, P6, P7 and P10) or comments from others (P4 and P5).

Over the period of time that um [pause] [sigh] I started to realise. Because you can see, you tell by your clothing, you can tell by your belt…tight trousers. …It just feels uncomfortable to me ‘cause I’m not used to having this extra weight on. (P1)

### Ground to a halt

Participants described being pinned down by physical and psychological weight.

Well the whole situation…of being mentally ill and then being made overweight… then having medicine that made me feel ill and overwhelmingly not quite myself, not myself at all. It really devastated me, it changed my life. (P2)

#### Losses upon losses: Losing control, losing self‐worth and losing myself

Weight gain compounded the negative impact of psychosis on self‐concept. Participants already felt that their confidence had taken a hit. Now weight gain, self‐perceived degraded appearance and, to a lesser extent, reduced fitness, posed another blow.

The experience of uncontrollable, stealth weight gain left participants with a sense of being ‘out of control’. This was one of many losses. Participants described losing the motivation to take care of themselves, losing confidence, losing connection and, finally, losing hope. This sense of loss, and specifically loss of identity, was central to all participants’ accounts.

Then you look different and you look in the mirror and you feel different about yourself … those months were where it started to become slightly distressing… A bit upsetting and very, very damaging to my sort of ego and self‐esteem. (P8)

If you’re not pleased with yourself, with the way you look, with the way you are, that’s gonna show in your social life and all aspects of life… If you’re not happy with yourself or with your current life situation then it’s gonna show in all other areas. (P4)

Weight gain led many to compare themselves unfavourably to others. There was already a sense of ‘not fitting in’ due to experiences of psychosis, but this was exacerbated by appearance concerns. The key themes were being changed, different and worse.

I didn’t feel as good as other girls my age… ‘cause I felt that they were slim and attractive, and I was cumbersome…and not very attractive because I was so overweight. (P2)

#### Amplified vulnerability – appearance, stigma and psychotic experiences

When you have psychosis, things can become a bit of an echo chamber… I would have thought is it because I’m, I’m, I’m a big guy now that, that, that person said that or that person thinks that?… I will, I’ll sort of worry that there’s a hidden meaning. (P8)

Participants faced major changes in their appearance. All participants experienced weight gain and more than half gained several stone. Participants described the excess weight as ‘cumbersome’ (P2), ‘disgusting’ (P3) and ‘unattractive’ (P9). This resulted in stretch marks and reduced options for clothing: ‘I couldn't really wear the kind of clothes that I used to wear before I went into hospital; smart, modern, up‐to‐date clothes. I had to wear whatever I could get into…and they were kind of frumpy things’ (P2). Appearance was specifically used as a metric for self‐evaluation: ‘Like, you know every day it's a bit of, it's a bit of a random check when you look in the mirror. You know do I feel happy about myself, do I not feel happy about myself?’ (P8). Appearance concerns were a source of distress, restriction, vulnerability and shameful difference. Participant 3 described his sense of self‐disgust, difference and shame.

Fear of negative evaluation from others was often based on experiences of stigma and fat shaming, leading participant 3 to conclude: ‘they just hate fat people’. Yet appearance concerns also fuelled unfounded ideas of persecution and threat: ‘I was more paranoid of people saying things, maybe judging me on my weight’ (P7). Often voices commented on appearance or food choices (P5, P7 and P9). Concerns about appearance led many to withdraw further.

I do get kind of anxious around people and I just assume they want me to leave, you know they want to get rid of me. You know that I’m disgusting. (P3)

#### Pulled in two directions – prioritising mental health

Tackling excess weight and achieving good mental health were both seen as important. Yet there was a major tension about timing and priority. For most participants, there was a clear focus to achieve a degree of stability in their mental health before addressing their weight. As participant 1 put it: ‘I’d rather be fat than crazy’. For others, there was no sense of urgency to address weight. Participant 4 described his initial view that tackling weight could be postponed. Yet, later discovered it was surprisingly challenging and frustratingly hard to shift. Three participants identified physical health problems such as pre‐diabetes and joint pain that they attribute to excess weight. While recognising the impact of weight gain on mood, confidence and activity, it was these physical health concerns that were driving their desire to address excess weight.

### An uphill struggle

So, before I got sick I would have been more inclined to go to the gym, to stick to a regime…go to the gym, eat healthier foods, care more about my appearance, care more about my physical health before I got sick…. I still go to the gym but I’m not as motivated. And that’s because of the illness, it’s because of what happened, it’s for sure. …Well, just lack of energy and that’s a mental, that’s like lack of motivation. I was like well, if I’m mentally unwell, if I can’t um be normal again what’s the purpose? I might as well get sick or die, I mean that, that’s my thought process. (P4)

#### The first step – regaining self‐worth

I wanted to feel like myself again. (P8)

There was a clear desire to reclaim a sense of normality. Tackling weight was seen as a key part of recovery. Yet the starting point for addressing excess weight is a diminished sense of self‐worth, self‐control and self‐efficacy. To begin this journey, participants needed to invest in rebuilding their self‐worth.

Self‐worth…once you’ve got enough of that…fully realised you are worth it. Like you do deserve to spend more time on yourself…to look after your body. I think that’s really important 'cause for a very long time I didn’t feel like I deserved anything…to be happy or healthy. (P3)

#### A heavy load

Participants worked hard to lose weight. Yet they faced additional burdens and hurdles. Participants spoke of the ‘shadow’ of psychosis. The ongoing presence of psychotic experiences such as voices or paranoia made it harder to engage in weight loss strategies, such as exercise: ‘I think gyms are sort of paranoid places in a way…. everyone goes and while you're there you think that everyone is watching you…’ (P7), or attending weight loss programmes: *‘*Um, it was scary yeah, it was scary, and I was paranoid, and my voices were kicking off’ (P10). This was compounded by the unintended sedative effects of medication, often making people feel intense fatigue, lack of motivation and rapid exhaustion on exertion: ‘I don't think many people on psychiatric medicine are motivated to do a lot of exercise. Because the medicine um that we're given slows us down’. (P2).

Beyond the specific psychotic experiences, participants faced challenges due to depression, withdrawal and rumination. Participants described feeling disconnected and isolated. Establishing social connection was even harder due to the double stigma of both psychosis and excess weight. With each additional hurdle, motivation took a hit.

When you don’t really see a future there’s no rationalising looking after your body ‘cause what is the point? If you’re depressed you don’t really think about the future you only think about what I’m doing now. (P3)

#### Maintaining momentum against the gradient

Participants described striving forwards only to feel pulled back. This led to frustration, resentment and despair. It was effortful to go against the gradient. Any success was often short lived. Regaining weight fuelled a sense of hopelessness. The lack of success was attributed to the necessity to continue taking obesogenic medication. There was a sense that losing weight was a losing battle.

Like when you’re up, down, up, down, up, down it’s like fighting a losing battle a little, but you know…And then the consequence of that is that you don’t fight the battle at all. And then the consequence of that is that you get fat. (P10)

Most participants struggled to achieve weight loss, and to identify preferences for treatment. Reconnecting and feeling understood, particularly through peer support, helped overcome the barriers of isolation and social discomfort, often helping to foster a sense of hope and agency.

You know that those people are going through the same experiences, so it makes you feel less isolated. The less isolated it makes you feel, the better, the better you feel, the more you can do. (P4)

Treatment approaches which are personalised and flexible (e.g., ensuring choice with regards to timing and support) were seen as empowering. Yet participants wanted expert input to set realistic expectations. Recognising that success perpetuates positivity and motivation. There was a desire for expert advice and accountability regarding targets but free from any sense of pressure or judgement. Participants acknowledged the tensions inherent in this request.

Looking back, participants wanted clear and frank information at the time of medication prescription.

I think [pause] if some more frank but nicely frank kind of conversations happened. If someone had said to me “you’re gonna put eight stone on in four months” I probably would have listened a lot more to their exercise plan. (P10)

There was a desire for specific information about expected weight gain, appetite changes and metabolic effects of medication. Yet acquiring knowledge was also seen as a potential route to mitigate the loss of control participants had experienced. Despite this, participants felt that tackling weight gain earlier would not have been possible due to the demands of coping with their psychotic experiences. Therefore, a combined and staged approach, incorporating peer support was preferred.

## DISCUSSION

This qualitative study set out to understand the experience of weight gain for patients with psychosis and their preferences for weight management interventions. The process of weight gain was described as a journey including multiple stages each with different psychological reactions and consequences. At first, weight gain came as a shock. It was experienced as yet another burden for patients to live with. Weight gain amplified a sense of vulnerability, which contributed to social withdrawal, depression and for some, psychotic experiences. The desire to lose weight was clear, yet the reality of achieving weight loss was tough and motivation fluctuated. Patients did not feel prepared for weight gain or supported to lose weight by professionals. Unsuccessful attempts at weight loss left little hope and few preferences for weight loss interventions. This understanding of the psychological journey may, however, offer potential novel targets for weight loss treatment development.

### Opportunities for intervention

The multiple stages in the journey of weight gain provide multiple opportunities for targeted intervention, which include: early, frank conversations to minimise weight gain; regaining confidence as a first step to weight loss; fostering hope by connecting with peers and experts‐by‐experience; setting realistic goals informed by weight loss and behavioural science and addressing the psychological impact, including a negative self‐concept, low mood and appearance concerns feeding into psychotic experiences. The current findings indicate that when developing weight management treatments consideration needs to be given to the timing, the target and the team who deliver it.

#### Timing

There is wide recognition that to address obesity, ‘prevention is better than cure’ (Pandita et al., 2016). Given the common timeframe of rapid weight gain for many patients with psychosis, there may be an opportunity for a preventative intervention. Yet in this study we found there was a double delay to starting weight loss efforts. At first weight gain was overshadowed by psychotic experiences and their treatment. Then, there was a lack of urgency, with participants noting that it was ‘not a life sentence’ and that it could be addressed later, if they wanted to. However, when it came to it, this was incredibly hard to achieve. Patients wanted early, frank conversations about common weight gain trajectories, informed by the differential effects of antipsychotic medications on weight gain trajectories (Perry et al., 2021; Pillinger et al., 2020). Resources to support these conversations have been developed (e.g., Orygen Youth Mental Health, 2016). However, it is likely that preventative efforts will need to be bolstered with later targeted treatments in recognition that physical health needs are often overshadowed by mental health problems in early psychosis.

#### Treatment targets

Standard behavioural weight loss treatments focus on weight change via calorie restriction, stimulus control (i.e., removal of environmental and behavioural cues, e.g., size of plates/portions and presence of high calorie foods) and increasing activity/calorie expenditure (Butryn et al., 2011; Wing et al., 2004). The model proposed in this study indicates novel treatment targets focused on the psychological consequences and processes within the weight gain journey. Most notable is the importance of regaining self‐worth and self‐efficacy in order to initiate and sustain weight loss. Although the relationship is likely bidirectional, as both depression and self‐esteem improve with successful weight loss interventions (Lasikiewicz et al., 2014), tackling self‐confidence may be necessary as a first step for patients with psychosis. A sense of vulnerability contributed to a number of other psychological consequences of weight gain including depression, inactivity, body image concerns, paranoia and hearing voices. Experiencing paranoid fears about threat from others or hearing negative voices commenting on appearance will understandably make it harder to engage in weight loss strategies. Interventions may also need to address issues concerning current societal stigma regarding both psychotic experiences and excess weight, with the potential to draw on the emerging body positivity movement ideas concerning more compassionate approaches. Given the ability to cope with distressing emotional and cognitive experiences plays a role in predicting long‐term weight loss success (Lillis & Kendra, 2014), interventions will need to account for these psychological factors (Rand et al., 2017).

#### Team

Within the NHS, there is a new workforce of experts‐by‐experience (NHS MH Long Term Plan Steering Group, 2019). Peer support workers are people with lived experience of mental health problems, who are trained to use their knowledge, experiences and empathy to support others receiving mental health services. The role varies by setting. Arguably working at the intersection of physical and mental health would be a valuable component of this role. The distinct expertise of peers and professionals has the potential to provide complementary approaches to weight loss. Connecting with peers and experts‐by‐experience may foster much needed hope. Interventions that include regular support by a practitioner with mental health expertise have been shown to be more effective (Lee et al., 2021). Interventions tackling excess weight will likely benefit from integrated support across multi‐disciplinary teams, including peer, medical and psychological expertise.

### A possible causal contributor to psychotic experiences

The physical consequences of weight gain are clear (Hayes et al., 2017). The impact on confidence and mood has been documented (Mccloughen & Foster, 2011; Tham et al., 2007). We have previously found associations between body image concerns and paranoia in the general population (Waite & Freeman, 2017) and in a qualitative study with patients experiencing persecutory beliefs (Marshall et al., 2019). We have shown that hearing voices commenting on appearance is common for patients seen in clinical services, with 50% reporting hearing daily negative content about appearance, for example ‘the voice(s) tell me that other people think I am fat’ (Waite et al., 2019). Voice content can mirror societal discrimination (Larøi et al., 2019). Given the double stigma described by participants, weight stigma may also contribute to the occurrence of mental health problems (Warnick et al., 2021). In the current findings, the contribution of weight gain to psychotic experiences is elucidated. In these accounts, weight gain contributes to the occurrence of psychotic experiences directly and indirectly via depression, social withdrawal and low self‐confidence. The current findings indicate potential mechanisms through which rapid weight gain may play a causal role in the occurrence of psychotic experiences. This highlights that weight gain may be an important treatment target for both physical and mental health.

### Limitations

There are several limitations of this study. First, the findings are not representative. None of the participants were interviewed during the first period of antipsychotic medication use most commonly associated with rapid weight gain. This may be particularly important when considering preferences for treatment. Nor was the sample representative in terms of diagnostic categories, and the most common diagnosis was psychosis NOS. Second, the findings indicate a potential causal route between weight gain and psychotic experiences; however, qualitative methods cannot be used to test causality. Third, the study was conducted within a research group using cognitive approaches to the understanding and treatment of psychosis. Despite efforts to minimise bias, such as multiple coders and validation with peer researchers with lived experience of psychosis, this will have shaped the analysis. Finally, few preferences for treatment were identified by participants. However, the proposed model indicates potential treatment targets for research.

### Future directions

The journey of weight gain in psychosis has multiple stages each characterised by different psychological reactions that may require distinct treatment approaches. The findings of this study provide a framework for exploring individuals’ experiences of weight change and preferences for treatment, including the timing of intervention, the specific focus of treatment and the team members involved. Weight management strategies that account for the specific experience and challenges of weight gain in the context of psychosis must now be developed and tested.

## CONFLICTS OF INTEREST

There are no conflicts of interest.

## AUTHOR CONTRIBUTION

**Felicity Waite:** Conceptualization (equal); Formal analysis (equal); Funding acquisition (equal); Supervision (equal); Writing – original draft (equal). **Amy Langman:** Data curation (equal); Formal analysis (equal); Investigation (equal); Project administration (equal); Writing – review & editing (equal). **Sophie Mulhall:** Data curation (equal); Formal analysis (equal); Investigation (equal); Project administration (equal); Writing – review & editing (equal). **Margaret Glogowska:** Formal analysis (equal); Methodology (equal); Supervision (equal); Writing – review & editing (equal). **Jamie Hartmann‐Boyce:** Methodology (equal); Writing – review & editing (equal). **Paul Aveyard:** Conceptualization (equal); Writing – review & editing (equal). **Belinda Lennox:** Conceptualization (equal); Funding acquisition (equal); Writing – review & editing (equal). **Oxford Cognitive Approaches to Psychosis Patient Advisory Group:** Conceptualization (equal); Formal analysis (equal). **Thomas Kabir:** Conceptualization (equal); Formal analysis (equal); Writing – review & editing (equal). **Daniel Freeman:** Conceptualization (equal); Formal analysis (equal); Supervision (equal); Writing – review & editing (equal).

## Data Availability

The data that support the findings of this study are not publicly available: due to privacy, it would not be ethically appropriate to share the whole data set. However, selected quotes to support claims made in the paper are available on request to the first author.
