# Supplementary material for: The psychological journey of weight gain in psychosis
Source: Psychol Psychother. 2022 Feb 8;95(2):525–40. doi: 10.1111/papt.12386 (PMC9304181; doi:10.1111/papt.12386)
Supplement: Supplementary file 1 [file PAPT-95-525-s001.docx]

Supplementary table 1. Illustrative participant quotes for each theme.

| Themes | Illustrative quotes |
| --- | --- |
| **Catapulted into obesity** | |
| Uncontrollable & rapid | (Pt 5) “It’s been two years. But in that time, I’ve, I’ve gained weight between being nine stone to 10 stone and that’s to do with tablets and eating all over the place.”  (Pt 4) “I stopped caring so much about my health and my weight… because I don’t care for my appearance, I’m gonna eat what’s unhealthy. So, the medication makes me hungry but the depression or the reality of the situation makes me make poor choices.”  (Pt 10) “I feel like the clozapine gave it a good kick start. But then I think I’ve got probably unhappy as well because of my weight so then it’s a vicious cycle from there on in.”  (Pt 2) “I wouldn’t be overweight if I wasn’t on this medicine. When I left hospital years ago, um, for the first four years I was admitted, I went from entering the hospital at eight stone and leaving at 13 stone.”  (Pt 10) “I just remember like there was always this hunger, it didn’t matter how much I ate kind of thing.”  (Pt 6) “I just don’t have that cut off when I’m full, I just eat, I would just eat food”  (Pt 4) “… the medication has an impact. It’s hard because…when you get hungry…it’s something you can’t control, and you can’t stop thinking about it. You keep thinking about food. So, I think it’s less of your responsibility, it’s something that has to happen maybe. You have to take the medication to be well but you might have to gain weight. And I see other people in reality and on the internet, I’ve seen them they gained weight, when they’ve had severe episodes they’ve all gained weight. So, it’s something that has to happen.”  (Pt 4) “I know my body has gotten used to the medication but at the beginning I was getting very hungry, so I was eating. So, it’s two reasons; it’s the medication in the beginning now my body has gotten used to it so I’m not that hungry.” … “I would say in the beginning 80-90% was just because of the medication.”  (Pt 8) “You cannot change it [weight gain] and suddenly it’s changed for the better or for the worse. And for most of the time I felt like it was for the worse.”  (Pt 1) “It was probably about five or six weeks er, I didn’t go out of the house a great deal.…because I didn’t want to go outside because of the psychosis…. It felt a scary place at the time.”  (Pt 8) “I didn’t really even have the motivation to do that even though I was unhappy with my weight. It felt like a lost cause almost. Maybe because I was thinking it was caused by the medication, I was thinking what’s even the point in trying to exercise off this weight? Because it’s just the medication and when I come off the medication it should be fine again.” |
| Stealth weight gain | (Pt 1) “I just wasn’t thinking about anything at that time, um, other than what I was going through with my psychosis… it wasn’t even on the radar to consider eating.”  (Pt 5) “It’s like your, your, your mindset wasn’t in the right place, so you didn’t think about your weight or how you were eating. It was just [pause] now, now that you’ve got you know you got better in time. Now you’re, you’re more aware  (Pt 6) “The weight wasn’t even on my mind… I was just out of it.” … “But I did notice when I was on the plane I had, they had to get me adjustable [extendable seatbelt]… I was a bit like, that’s when I realised, ‘shit’ you know… I was just like, ‘god’ you know [sigh] ‘I’ve gained like, I can’t believe how much I’ve gained’.”  (Pt 7) “My weight had increased by 100lb which I don’t know how it happened”  (Pt 7) “I dunno, I just lost control”… I was on like a full dose so, appetite was really high.” “I think it’s very easy to increase your calories and not realise... So I was just eating a lot more but didn’t realise it and I think my metabolism was increasing as I was eating more.”… “I was depressed but at the same time as having a high appetite. So, I didn’t care so much about whether I did put on weight.”  (Pt 8) “But at the time maybe I wasn’t too concerned about it… I was more concerned about the mental side than the physical….it was getting better mentally.”  (Pt 9) “… being mentally unwell like first like you’re not going to be able to look after your weight or anything.”  (Pt 9) “… from my experience like when I was really unwell with mental health, I, I didn’t look after, I wasn’t concerned like about um. I, I wouldn’t be concentrating on what I was eating… it’s because of the voices and [pause] and because the distraction… I couldn’t concentrate on things.” |
| Time lag | (Pt 1) “I’ve got no scales here or anything like that. Um, but it was just that…over the period of time that um [pause] [sigh] I started to realise. Because you can see, you tell by your clothing, you can tell by your belt.”  (Pt 5) “I hadn’t noticed, I hadn’t noticed my weight had changed. It was when [pause] when I thought, when I, I, I thought, ‘hold on a minute I’m getting, my husband’s now saying I’m getting bigger’… because I was ill I didn’t notice it.”  (Pt 10) “I wish someone, be that a doctor or my CPN or somebody had said something earlier. Because that, I clearly wasn’t recognising it [weight gain] in myself.”  (Pt 5) “Yeah, my family saw the weight gain and they obviously mentioned, they said, “you need to get his under control somehow”… “Well, that makes me feel more hopeless, more depressed”… “It’s that belief… of what’s going to happen about your health. It’s reaffirmed, it’s confirmed that other people are seeing it too ‘I am gaining weight, I am heading towards that direction of getting diabetes and heart disease’. So, when other people are telling you it has an impact, yeah.”  (Pt 7) “It went on quite quickly...but they didn’t they didn’t really mention it”… “They only mentioned it when I asked them”  (Pt 6) “Yeah, like you can’t wear what, wear clothes what, wear what clothes you want, and you’ve got to buy bigger sizes”  (Pt 7) “I only actually noticed when I saw the stretch marks in my arm… In my arms and in my legs and it was, it was like my skin was getting stretched, and I thought it’s getting a bit much.”  (Pt 7) *“*I think the big lightbulb moment where I realised I had to lose weight was when I’d test myself physically and realised that I was sort of declining in terms of physical fitness. Like just going for walks, jogs, walking up hills… Realising that putting all this fat on, it’s made me a lot less fit. … I just thought how did I get this unfit? ‘Cause it happens very quickly”  (Pt 4) “I mean it’s serious but it’s not that serious. I mean I, I can make the decision to change this at any time. I can start eating healthier and it’s not like I’ve got [pause] it’s not like it’s a consequence, it’s a definite consequence. It’s a I can change that at any time so it’s not that I don’t know what’s the word? It’s not a life sentence the weight gain.” |
| **Ground to a halt** | |
| Losses upon losses | (Pt 2) “I didn’t feel like a normal person anymore. I knew I was different from the others and I didn’t like [it]”  (Pt 2) “… it made me feel less confident… I didn’t feel attractive any more, I felt very unattractive and cumbersome and big.”  (Pt 7) “I wasn’t too angry… Just being a bit disappointed in myself”  (Pt 1) “The stone, extra stone made a difference. And I need to, I need to get rid of it um to improve, improve or get back to where I was… It just feels uncomfortable to me coz I’m not used to having this extra weight on.”  (Pt 4) “… like you’re already mentally unwell, well you’re taking medication, do you want another illness you know like diabetes or heart disease?”  (Pt 8) “[Interviewer]*What was distressing about … (the weight gain)?”…* **“**not having the [pause] the body that I, I almost felt like I was meant to have.”  (Pt 6) “That time when I was a write-off… my friends didn’t invite me out… I just literally just withdrew from society… I was just so out of it like.”… “When I was lighter I’d go out more, I’d like go for the shop I’d go walk about and stuff… just because…. I had more motivation. Like if you’re heavy you don’t want to move coz it’s like harder to move.”  (Pt 2) “Well, the whole situation… of being mentally ill and then being made overweight… And then having medicine that also made me feel ill um and overwhelmingly not quite myself, not myself at all. Um, it really devastated me, it changed my life”  (Pt 2) “I think it was linked to the fact that um, [pause] subconsciously I realised I wasn’t the same person as I, as I was when I was, or as I should have been or could’ve been er when I was younger. Because of the medicine, the weight gain and the medicine er because I didn’t want to be so active um. When I came home from work, the first job I had after leaving hospital I used to go to bed after I’d had my dinner and I’d just sleep till morning. And my poor mum didn’t know what to do with me, she said, “you’re not living [name], you don’t, you need to get out and meet people” and I didn’t really want to. I was so depressed.”  (Pt 2) “No, well I didn’t feel normal, I didn’t, I didn’t feel like a normal person anymore. I knew I was different form the others and um I didn’t like, didn’t like feeling different from the others.”  (Pt 4) “As soon as my other aspects of my life, my getting a job… didn’t go very well… So, like I said for me those are very important. So, as soon as the decline of, of pursuing I don’t know educational or professional goals. As soon as that stopped my desire for improving my weight also stopped.”  (Pt 4) “… well I would say when you suffer from a severe mental health problem um you feel like [pause] you’re not normal. I, that’s how I feel, I feel like I’m not normal anymore.”  (Pt 6) “… it was just, I was a complete write off like.”  (Pt 10) “It’s more about like I guess for me because sports was my everything, I guess there’s a little bit of me that’s like don’t wanna do it if I can’t do it properly.” |
| Amplifies vulnerability | (Pt 3) “I had an image, I could not change it. I was like disgusting, repulsive hideous, evil you know and that was just in my head what I was.”  (Pt 2) “I didn’t feel attractive anymore, I felt very unattractive and cumbersome and big um. I, I could see I was overweight”  (Pt 2) “No, well I didn’t feel normal, I didn’t, I didn’t feel like a normal person anymore. I knew I was different form the others and um I didn’t like, didn’t like feeling different from the others.”  (Pt 9) “[Interviewer]*Why would voices comment on weight/appearance?”… “*I think like it’s just to make me feel even worse. Because they knew probably like weight was something that I always concerned about.”  (Pt 10) “If there was a threat I can’t get away at the minute. Which is probably why I’m more overly conscious of where the threat’s coming from like. Whereas I think when I was younger although the thought was there I was like well, if someone comes I’ll just run.”  (Pt 7) “I think a lot of society is appearance focused … I think even if like they might not say it, they might just think it.”  (Pt 9) “And they, they’re not going to be happy to see me like, they will not want me.”  (Pt 3) “I’ve never felt good eating in public, er I wouldn’t really. … just feel like people are looking at me and like laughing at me.”  (Pt 3) “I do get kind of anxious around people and I just assume they want me to leave, you know they want to get rid of me. You know that, that I’m disgusting.” |
| Pulled in two directions | (Pt 9) “It’s better being fat than crazy!”  (Pt 4) “I was still preoccupied by my physical appearance, to a lesser extent obviously I had other things going on obviously.”… “I mean you’re still, it’s, it’s, you, you’re living two realities. It’s your reality of being crazy [laughing] and it’s your other reality where it’s the actual reality where you’re, you’re still overweight.”  (Pt 7) “I’ve always sort of been bothered about it [my weight]… that never ceased… It’s just mental health was number one in the ranking. … And then as my mental health was somewhat improved to the point where I was able to work, able to drive, physical health began to be up there too.”  (Pt 10) “… you can take this [Antipsychotic medication] and put some weight on but feel better or you can stay as you are”.  (Pt 9) “I think like being healthy means like either you’ve got to be mentally healthy and physically healthy as well. … Being mentally unwell like first like you’re not going to be able to look after your weight or anything. From my experience like when I was really un, unwell with mental health I, I didn’t look after, I wasn’t concerned like about um. I, I wouldn’t be concentrating on what I was eating you know, so… because of the voices and [pause] and because the distraction as well. Like you wondered, like I say like I couldn’t concentrate on things so yeah… But I think a few months after [clears throat] should starting the medication that would be helpful.”  (Pt 2) “… the weight um was all part of, of it, it was all part of it. I mean I, I felt that um, I mean I did not take the drugs, I stopped taking them once or twice in my life. Because I, I thought that you know I’d be able to get a job and I’d be able to do without them, the drugs. But unfortunately, um and after, about a month after I took the drugs, stopped taking the drugs I mean, I got mentally ill again. But I did go down to eight stone and after staying off of them just over a year, I went down to eight stone again.” |
| **An uphill struggle** | |
| The first step | (Pt 1) “You open yourself up to the world again and then start returning to normality…. By saying right, now I need to start doing things that are good for me.”  (Pt 9) “I want to feel good about myself. And I want to prove that wrong that I could feel good and I could look good and er [pause]. I think that motivated me most.”  (Pt 3) “As long as it’s fully rationalised, fully realised [that] you are worth it. Like you do deserve (kind of) to spend more time on yourself… to look after your body. I think that’s… really important coz for a very long time I didn’t feel like that, I didn’t feel like I deserved anything. Like I don’t deserve to be happy or healthy…[so] I don’t do anything to make it that way.”  (Pt 4) “A big part is also me, because I have to want it. So, those are all aids that can facilitate the, the process of improvising my weight, but it ultimately comes down to me to taking control” … “I guess if my overall life situation improves then I would feel like I would be able to take control. So…I need…for other aspects of my life to improve. I need something to go well in my life so then I can get the ball rolling you know.”… “I know when other areas of my life are going well, I know if my life improves so the motivation keeps coming back.” |
| Heavy load | (Pt 1) “I’m now back up to mountain biking, swim, running and um [pause] mountain biking, running and gym. And it doesn’t seem like it’s shifting at the moment….yes, frustrating.”  (Pt 4) “I would compare my life to their life and that would make me feel a bit more depressed. Like I wish I was like him, I wish I was healthy, mentally healthy.”  (Pt 7) “Stopped going to the gym for a period...paranoia, anxiety, [worried about being] judged mainly. That was a big one for me.”  (Pt3) Er no it’s just, it, it’s not that it doesn’t feel that way it, it flat out isn’t true. They just hate fat people but that, that’s the way [laughing]… people will, they will rationalise its they are trying to help you know the way they see it. [Pause] you know people are unhealthy, they’re unhappy, they’re not happy like I am. But yeah, I, I don’t buy it, they [laughing].  (Pt 4 ) “I’m not as interested in people because I feel like I don’t have much to, not to offer but I’m just not really as, as motivated. It’s again, it’s for every aspect it’s like, it’s motivation to do those things it’s. Even to interact with other people you have to be a little bit interested and motivated to engage in.”  (Pt 5) “Your mindset isn’t there properly, you know your, you get up. You, you, you don’t function, you’re like, you’re like a robot in a way. Um, it’s, it’s like you, you, you’re on a, you’re on a treadmill and you’re just this robot that’s getting up every morning, do, doing you know trying to get the children to school. But you are still poorly, you’re still poorly yourself.” |
| Keeping momentum against the gradient | (Pt 7) “I remember going to the gym when I re-joined the gym in town, started going again I was really paranoid at first… But and I still sort of am but I’ve learnt to just get on with it and enjoy it for what it is.”  (Pt 9) “If I’m by myself (at the gym) I’m just thinking and then worrying about things. But if you are with someone or distracted with other things and talk about other things.”  (Pt 10) “I’m on with kind of a like a peer role where someone has kind of been through it themselves. Basic stuff but more just about, I guess about “look, I get it, it’s tough you know I’ve been there”.  (Pt 8) “To see them going out and doing it, definitely encouraged me to do it.”  (Pt 8) “… if someone had sat me down and said like, “people do gain a lot of weight on this and you know that’s not a healthy thing… I would have been a bit more mentally prepared for it.”  (Pt 7) “Maybe talking from early, like with the very first consultant. And actually said, coz they say, “you might put on a bit of weight” and I was thinking in my head you know 10 lb. But it’s actually, it’s actually a lot more than that you end up putting on.”… “Yeah, it might help to and a real talk about “you’re going to have to stay in control of your diet”.  (Pt 7) “more the social aspect…going and meeting all these new people might make me feel a bit uncomfortable at first so I sort of avoid it.”  (Pt 2) “I think that um [pause] getting like-minded people with things in common, that are mentally ill does help motivate… Because to, to try to um lose weight um, er [pause] I mean people in groups are, do things more easily than. Especially if they’ve got a problem like … non-motivation medicine… a group of people will find it easier with with a… teacher or a helper.”  (Pt 4) “Maybe for me what would help if I had a gym partner… So, again that goes back to if you had a support group where you, everybody meets and they workout. So, if I had a partner to go to the gym that would help as well, um. And again, that ties with the social interactions you know, if you have friends and whatever it’s easier to maybe you go to the gym together, you know you cook some meals together. You keep yourself accountable”  (Pt 1) “I’m now back up to mountain biking, swim, er running and um [pause] mountain biking, running and gym. And it doesn’t seem like it’s shifting at the moment… yes, frustrating.”  (Pt 7) “[Medication plays a] massive role, increase in appetite also, think there’s something, I guess you know that like olanzapine does in terms of the way you metabolise, well the way you digest sugars is it?... or it makes it um easier to put on weight, harder to lose weight.”  (Pt 2) “Since I’ve been on um the psychiatric medicine and that is the cause of it. And I said if I had um a better um medicine to take that would enable me not to put on the weight I wouldn’t be so, I wouldn’t be as the way I am.”  (Pt 10) “I know what, know what I need to do and every time I’m well I’m gonna take the opportunity to do that. And then when I’m ill I’m gonna try and do the least amount of damage I can [laughing].” |
