# Supplementary material for: The psychological journey of weight gain in psychosis
Source: Psychol Psychother. 2022 Feb 8;95(2):525–40. doi: 10.1111/papt.12386 (PMC9304181; doi:10.1111/papt.12386)
Supplement: Supplementary file 2 [file PAPT-95-525-s002.docx]

Supplementary materials.

**Topic Guide- Exploring patient experiences of weight change**

This interview schedule is intended to be used flexibly to facilitate eliciting the patients’ own account of their experiences of weight changes and preferences for interventions to promote healthy weight or prevent weight gain. There are four main topic areas. Example questions are given for each of the main topic areas. To promote engagement, the participants own language will be used whenever possible.

Introduction: We want to find out more about your experiences and views about your weight, and especially about what you think about any treatments for changing your weight.

1. Experience of weight
   - Could you tell me about your experience with your weight? Have there been any changes?
   - How do you understand any changes in weight?
2. Impact of weight
   - Is weight something that is important to you?
   - In what ways does weight impact your life? Does your weight affect your wellbeing?
3. Satisfaction and effort to change weight
   - Is there anything that makes you want to change your weight?
   - How important is managing your weight to you?
   - Have you ever thought about or tried to manage your weight/ reach your preferred weight?
4. Preferences for treatment
   - What would be your preferences for interventions to promote healthy weight? *(in relation to prevention of weight gain and interventions for weight management)*
5. Further comments
   - Is there anything we have not covered that you would like to share?
